# Evaluation of White Grape Marc Extract as an Additive to Extend the Shelf-Life of Fish Fillets

**DOI:** 10.3390/foods14081438

**Published:** 2025-04-21

**Authors:** María Isabel Sáez, Javier Sabio, Alba Galafat, Antonio Jesús Vizcaíno, Francisco Javier Alarcón-López, Tomás Francisco Martínez Moya

**Affiliations:** Departamento de Biología y Geología, CEIMAR, Universidad de Almería, 04120 Almería, Spain

**Keywords:** agrifood by-products, antioxidants, fish preservatives, gilthead seabream fillets, grape marc extract

## Abstract

In this study, an extract of white grape marc (GME), a by-product obtained during the winemaking process, was applied to the surface of gilthead seabream (*Sparus aurata*) fillets, which were then stored under refrigeration (4 °C) for a period of 14 days. The effects of GME were compared with those of ascorbic acid (one of the few additives authorized for fresh fish in the EU) and distilled water (as a control batch). Samples were taken at 1, 2, 4, 7, 9, 11, and 14 days postmortem (dpm) cold storage, and several objective quality parameters were measured (instrumental color, pH, water holding capacity, texture profile analysis—TPA, lipid oxidation, and microbial spoilage). The results showed that the grape extract significantly improved several of the parameters studied, not only compared to the control batch, but even compared to the ascorbic acid batch. Thus, GME slowed down the proliferation of psychrophilic bacteria, prevented the oxidation of muscle lipids, and even improved the firmness of the fillets compared to the other two experimental groups. On the other hand, minor effects on color, pH, or water retention capacity were observed. In the context of the scarcity of approved food additives for fresh fish in the EU and the strong consumer rejection of synthetic substances for this purpose, this grape extract could well represent a viable alternative. In addition to its natural origin, the use of GME as a food additive could also contribute to the valorization of winery by-products as part of a circular bioeconomy strategy.

## 1. Introduction

Agri-food industries generate millions of tonnes of by-product waste, a scenario that has led to a focus on developing strategies to minimize the negative environmental impact of these industrial residues. In this context, recycling is emerging as an innovative strategy to improve the sustainability of the production chain, and to promote the commercialization of novel natural components, encouraging the creation of initiatives for their extraction, transformation, and commercialization [1,2]. The wine sector represents an important agri-food activity in the European Union (EU), which is the world’s leading wine-producing region, accounting for 7.5% of the total value of agricultural production. Around 3.2 million ha are cultivated [3], representing 2% of the EU’s agricultural land, and around 60% of the global vineyard area. This land is distributed over several countries, the most important being Spain, France, and Italy, which together account for more than 70% of the total EU vineyard area.

Wine production consumes more than 80% of the grapes harvested [4], and generates a significant volume of by-products, mainly grape marc, the residue remaining after pressing the fruit. Raw grape marc is a complex biomass that includes elements such as stems, skins, seeds, and residual pulp [5,6], and represents approximately 30% of the original weight of the grape. This huge amount of by-products can become an economic and environmental issue unless it is managed properly [1,4]. Among the great variety of uses of grape marc [6], it is worth mentioning its inclusion as an ingredient in animal feeds [7,8], including farmed fish [9,10,11], with these studies reporting overall beneficial effects on several physiological aspects of fish fed on this biomass. The development of innovative bioactive food packaging materials derived from grapple marc can also be cited, which not only helps to reduce plastic usage but also contributes to preserving food quality [12,13].

On the other hand, the use of authorized additives to extend the shelf-life of fresh fish is severely restricted in the EU, as synthetic antioxidants have been associated with health issues due to their potential toxicity [14]. Therefore, only citric and ascorbic acids and their salts, are permitted for this purpose (Regulation (EC) No. 1333/2008, on food additives [15]). As a result, the search for sustainable and cost-effective alternatives based on additives of natural origin (which are much more acceptable to consumers than those of chemical synthesis) is currently an urgent need for the food industry [2]. Among the potential natural preservatives investigated in the last decade, not only for their antioxidant potential but also for their antimicrobial properties, those derived from aromatic plants rich in phenolic compounds stand out, such as extracts from cinnamon, basil, garlic, oregano, rosemary, and thyme, all of which have shown valuable results in fish [16,17]. Many bioactive components have been described in grape marc, including several extractable phenolic antioxidants such as flavonoids, phenolic acids, procyanidins, and resveratrol [18,19]. The distribution of total extractable phenolics is not uniform in all fruit fractions, with approximately 60% of them found in the seeds, 30% of them found in the skin, and the remainder (10%) in the pulp [6]. These molecules may be of interest in different areas of the food industry for use as food additives, due to their known antioxidant and antimicrobial properties, but especially in the preservation of fresh fish, given the scarcity of substances approved for this purpose. In this regard, very few studies have addressed the use of grape marc extracts for direct treatment of fillets, and thus, Sánchez-Alonso et al. [20] evaluated their potential on horse mackerel fillets and observed an increase in their shelf-life due to a reduction in the oxidation of muscle lipids.

Based on the abovementioned antecedents, the initial hypothesis of this study is that grape marc extract may have favorable effects on the preservation of refrigerated fish for human consumption, extending their commercial shelf-life and quality attributes, compared to the untreated product. In order to evaluate this hypothesis, the objective of this study was to assess the effects of commercial grape marc extract (GME), used as an external treatment, on the quality and shelf-life of refrigerated gilthead seabream (*Sparus aurata*) fillets, one of the most important species in the Mediterranean aquaculture. Possible effects on the instrumental quality parameters and shelf-life will be compared with two other experimental groups: on the one hand, untreated fillets (control group) and, on the other hand, fillets treated with the reference additive authorized for fresh fish (ascorbic acid, ASC).

In this way, the study will investigate whether this by-product can be used as a functional and sustainable additive for fresh fish, thereby adding value to it by exploiting its bioactive properties and characteristics, while at the same time contributing to and promoting the circular bioeconomy.

## 2. Materials and Methods

### 2.1. Additives

An aqueous dilution (0.03% *v*/*v*) of a commercial extract from *Vitis vinifera* white grape marc (i-Grape grape extract^®^ 500 mL, i-Grape Laboratories, Santiago de Compostela, Spain) was prepared using sterilized water (grape marc extract batch, GME). An aqueous solution (1% *w*/*v*) of ascorbic acid (Sigma Aldrich, Madrid, Spain) was also prepared for comparative purposes (ASC batch).

### 2.2. GME Total Phenolic Content

Folin–Ciocalteu spectrophotometric procedure was carried out as described by Singh et al. [21]. A gallic acid (Sigma Aldrich, Madrid, Spain) standard was prepared (0 to 200 μg mL^−1^), and the results for total phenolic content were expressed as mg of gallic acid equivalents mL^−1^.

### 2.3. GME Ferric Reducing Antioxidant Power (FRAP)

The antioxidant capacity of the grape extract was estimated according to the methodology described by Hajimahmoodi et al. [22]. Working solutions were prepared by mixing 100 μL of the samples or standards with 3 mL of FRAP reagent (50 mL 0.3 M acetate buffer, pH 3.6, 5 mL 10 mM tripyridyltriazine prepared in 40 mM HCl, and 5 mL 20 mM FeCl_3_), and kept in the dark for 20 min at room temperature. Absorbance was then measured at 593 nm. Standards were prepared with ethanolic solutions of 6-hydroxy-2,5,7,8-tetramethyl-chroman-2-carboxylic acid (Trolox; Sigma Aldrich, Madrid, Spain), and the results were expressed as μmol Trolox equivalents mL^−1^.

### 2.4. GME Radical Scavenging Activity Determination (DPPH)

This activity was measured according to the method described by Brand-Williams [23]. The reaction mixtures were prepared by adding 75 μL of the grape extracts into 150 μL of 100 μg mL^−1^ 2,2-diphenyl-1-picryl-hydrazyl-hydrate (DPPH) solution, and then incubated at room temperature in the dark for 24 h. The transformation of DPPH from oxidized to reduced form was determined spectrophotometrically at 515 nm. Standards were prepared with ethanolic solutions of Trolox. Results were expressed as μmol Trolox equivalents mL^−1^.

### 2.5. Experimental Design

A total of 42 gilthead seabream fish of commercial size (300 ± 20 g) were used. The fish were filleted within two hours after slaughtering, and the obtained fillets (n = 84) were divided into 3 experimental groups, each containing 28 fillets (corresponding to 7 sampling times × 4 fillets per sampling time), as follows: (i) CT, fillets treated with autoclaved distilled water; (ii) ASC, fillets treated with a 1% ascorbic acid solution; and (iii) GME, fillets treated with a 0.03% (*v*/*v*) grape marc extract.

The fillets were placed on racks (7 fillets per rack), previously sterilized by dry heat, and the respective solutions were uniformly applied to the fillet surfaces using a high-pressure low-volume (HPLV) air gun device, equipped with a 50 mL capacity reservoir and a nozzle size of 0.3 mm. The fillets were treated on both the skin and muscle sides with the respective sprayed solutions for 30 s (approximately 10 mL per fillet), left to dry for 5 min, then packaged in sterile transparent polyethylene bags and stored in a refrigerated chamber (4 ± 1 °C). Samples were taken at 1, 2, 4, 7, 9, 11, and 14 days postmortem (dpm) for the following analyses: psychrophilic microorganisms counts, pH, water-holding capacity (WHC) of the muscle, texture profile analysis (TPA), skin and muscle color, and muscle lipid oxidation status.

### 2.6. Total Viable Counts

Total viable psychrophilic bacteria counts were carried out according to Sáez et al. [24] on fillet samples up to 14 dpm. Briefly, the anterior dorsal muscle of fillets was cut into cubes (1 g), transferred aseptically into sterile tubes containing 10 mL of 0.1% (*w*/*v*) peptone water (Cultimed, Madrid, Spain), and homogenized for 60 s using a sterile mechanical homogenizer (Polytron PT-2100, Kinematica AG, Lucerne, Switzerland). Total viable psychrophilic bacterial counts were quantified using plate count agar (PCA, Merck, Darmstadt, Germany), after incubation for 120 h at 4 °C. Microbiological loads were expressed as logarithm of colony-forming units (cfu) per gram.

### 2.7. Lipid Oxidation

Lipid oxidation was estimated by thiobarbituric acid-reactive substances (TBARS) analysis throughout the 14 d storage period. TBARS were measured in muscle samples according to the spectrophotometric method of Buege and Aust [25], as detailed in Molina et al. [26]. The amount of TBARS was expressed as mg of malonyl dialdehyde (MDA) per kg of muscle after comparing it with an MDA standard.

### 2.8. pH and Water Holding Capacity (WHC)

Flesh pH was determined in the anterior part of the dorsal muscle by means of a penetration electrode (Crison, Barcelona, Spain, model GLP 21; sensitivity 0.01 pH units) as described in Suárez et al. [27]. WHC (expressed in percentage) was calculated from a piece (1 cm^3^) of the anterior part of dorsal muscle as the difference between the initial percentage of water and the percentage of water released after centrifugation, as detailed in Suárez et al. [27].

### 2.9. Texture Profile Analysis (TPA)

Fillet texture profile analysis (TPA) was measured by compression of the anterior area to the dorsal fin, above the lateral line of fillets using a Texture Analyzer (TXT2 plus “Stable Micro System”), equipped with a load cell of 5 kN, controlled with Texture Expert Exceed 2.52 software (Stable Micro Systems, Surrey, UK). Muscle samples were subjected to two consecutive cycles of 25% compression, with 5 s between cycles, in which a 20 mm cylindrical probe was used for pressing downwards into the fillet at a constant speed of 1 mm s^−1^. The textural parameters hardness, springiness, cohesiveness, gumminess, chewiness, and resilience were calculated as described in Bourne [28].

### 2.10. Instrumental Color

Fillet muscle color was measured thrice by L*, a*, and b* system [29], using a Minolta Chroma meter CR400 device (Minolta, Osaka, Japan). The lightness (L*, on a 0–100 point scale from black to white), redness (a*, estimates the position between red, positive values, and green, negative values), and yellowness (b*, estimates the position between yellow, positive values, and blue, negative values) were recorded.

### 2.11. Statistics

The effect of the categorical variables “additive” and “storage time”, as well as their interactions, were determined for each numeric parameter studied by fitting a generalized linear statistical model (SPSS 23, IBM Corporation Inc., Armonk, NY, USA). Least squares means were tested for differences using Fisher’s LSD procedure. Data expressed as a percentage were normalized by arcsine transformation of the square root prior to the analysis. A significance level of 95% was considered to indicate statistical difference.

## 3. Results

### 3.1. Grape Marc Extract (GME) Total Phenolics and Antioxidant Activities

Total phenolic contents (TPC) of GME expressed as mg of gallic acid equivalents per mL, ferric reducing antioxidant power (FRAP), expressed as μmol equivalent of Trolox per mL, and radical scavenging activity (DPPH), expressed as μmol equivalents Trolox per mL, in commercial GME are shown in Table 1.

### 3.2. Total Viable Counts

**Figure 1:**
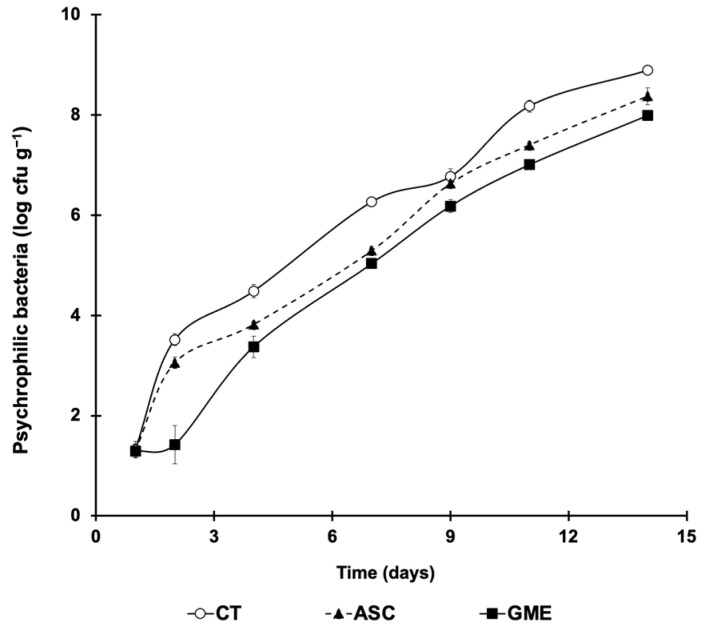
Changes in psychrophilic bacteria counts (log cfu g^−1^) in fillets throughout the 14 d cold storage (4 °C) period. CT: control. ASC: ascorbic acid batch. GME: grape marc extract. Values are expressed as mean ± s.d. (n = 4 fillets per dietary treatment and sampling time).

Psychrophilic bacteria total viable counts (TVC) in fillets throughout the cold storage period are shown in Figure 1. Storage time (*p* ≤ 0.01) and additive treatment (*p* ≤ 0.01) as well as their interaction (*p* = 0.02) caused significant effects on this parameter.

The results indicate that, as storage under refrigeration progresses, TVC increased in all fillets, but at different rates depending on the experimental batch. It is notable that the two additives, GME and ASC reduced TVC values compared to untreated fillets (CT). After only two dpm TVC showed significant differences among treatments, with lower values in GME fillets (1.42 log CFU/g) compared to CT and ASC fillets (3.51 and 3.05, respectively). However, from 4 dpm onward, GME and ASC values were similar to each other, but still significantly lower than those obtained in CT, a tendency that persisted up to the end of the assay (14 dpm).

### 3.3. Lipid Oxidation

**Figure 2:**
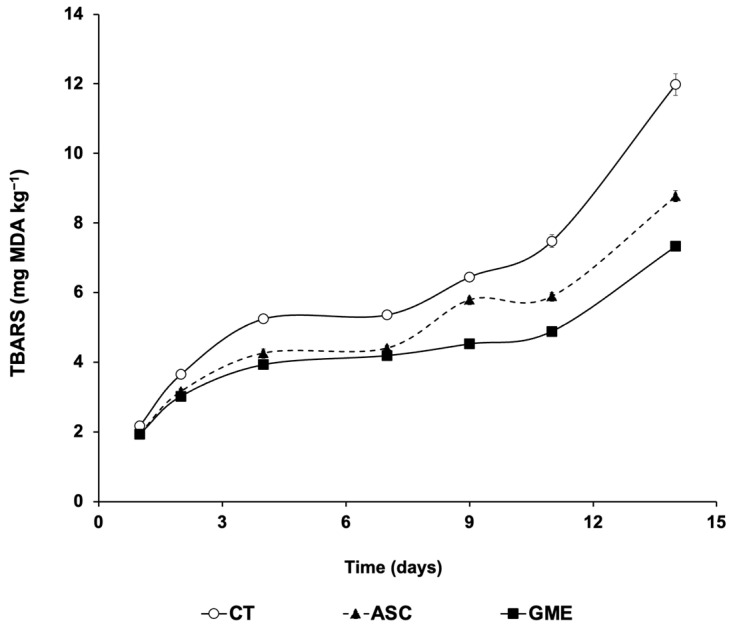
Influence of the experimental treatments on postmortem changes in thiobarbituric acid reactive substances (TBARS) in fillets stored under refrigeration (4 °C). Results are expressed as malonyl dialdehyde (MDA) equivalents (mg kg^−1^ muscle). CT: control, ASC: ascorbic acid, GME: grape marc extract. Values are expressed as mean ± s.d. (n = 4 fillets per dietary treatment and sampling time).

The time course of postmortem changes in muscle lipid oxidation of fillets, as determined by thiobarbituric acid reactive substances (TBARS) content, is shown in Figure 2.

Roughly, lipid oxidation was influenced by storage time, additive treatment, and their interaction (*p* ≤ 0.01 in all cases). TBARS values increased with storage time, although both ASC and GME fillets clearly protected muscle lipids from oxidative deterioration throughout the entire storage period compared to the CT batch (*p* ≤ 0.001). Within the additives, no differences were observed for ASC and GME up to 7 dpm, but from this time point onward, fillets treated with GME yielded significantly lower MDA equivalents than those from the ASC lot.

### 3.4. Fillet pH and Water Holding Capacity (WHC)

**Figure 3:**
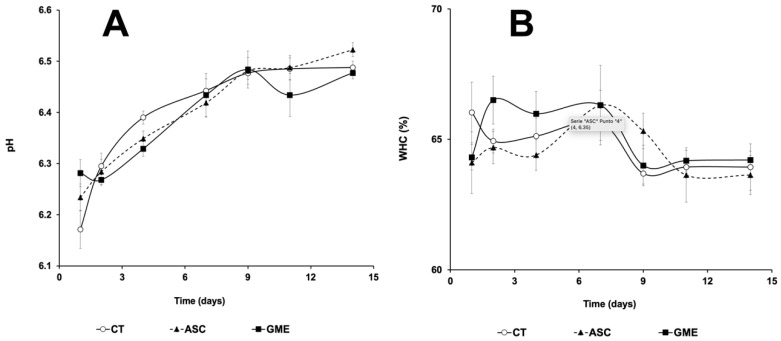
Influence of the different treatments on postmortem changes in pH (**A**) and WHC (**B**) of gilthead seabream (*Sparus aurata*) fillets throughout the cold storage period at 4 °C. CT: control, ASC: ascorbic acid, GME: grape marc extract. Values are expressed as mean ± s.d. (n = 4 fillets per dietary treatment and sampling time).

Changes in pH (A) and WHC (B) during 14 days of cold storage of fillets are shown in Figure 3. The factor storage time was responsible for the increase in pH values of fillets (*p* ≤ 0.01), whereas no differences attributable to the additives were observed.

For WHC (Figure 3B), no clear differences were observed among the three experimental batches, and none of the factors studied (storage time, antioxidant treatment, or their interaction; *p* = 0.752, *p* = 0.310, and *p* = 0.096, respectively) caused changes in this parameter.

### 3.5. Fillet Texture Profile Analysis (TPA)

**Figure 4:**
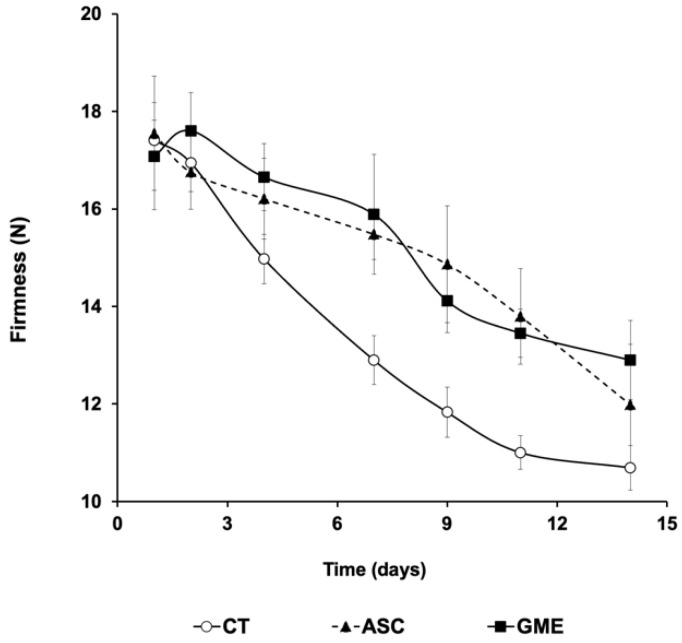
Influence of different antioxidant treatments on postmortem changes in firmness of sea bream (*Sparus aurata*) fillets during 14 days of cold storage at 4 °C. CT: control, ASC: ascorbic acid, GME: grape marc extract. Values are expressed as mean ± s.d. (n = 4 fillets per dietary treatment and sampling time).

The influence of the treatment of sea bream fillets with the additives on the textural parameters is summarized in Figure 4 and Table 2.

The parameter firmness (Figure 4) decreased in all treatments during the storage time. However, from 4 dpm onward, the CT fillets showed significantly lower values than those treated with the additives, and these differences were maintained at the end of the experimental period (day 14). When comparing the GME and ASC batches, there was no statistical difference between them over the entire shelf-life.

With regard to the other TPA parameters (Table 2), overall, gumminess and chewiness showed higher values for both ASC and GME compared to the CT batch, although differences were not significant for all the sampling points. The parameters springiness, cohesiveness, and resilience showed no clear trend or differences among the experimental groups.

### 3.6. Instrumental Color

**Figure 5:**
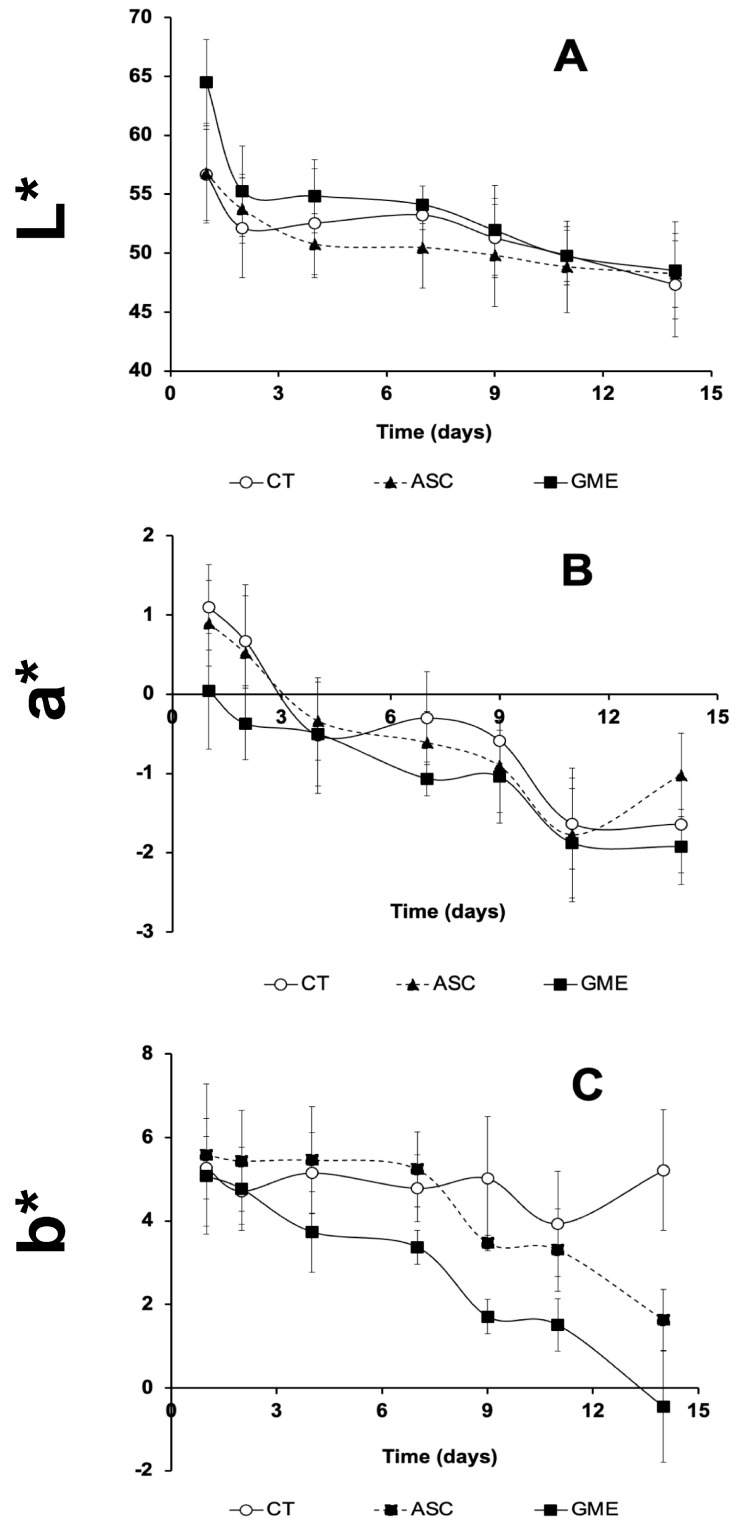
Time course of changes in chromatic parameters ((**A**): lightness, L*; (**B**): redness/greenness, a*; (**C**): yellowness/blueness, b*), of skin side of gilthead seabream fillets treated with the different experimental solutions during 14 days of cold storage at 4 °C. CT: control, ASC: ascorbic acid, GME: grape marc extract. Values are expressed as mean ± s.d. (n = 4 fillets per dietary treatment and sampling time).

**Figure 6:**
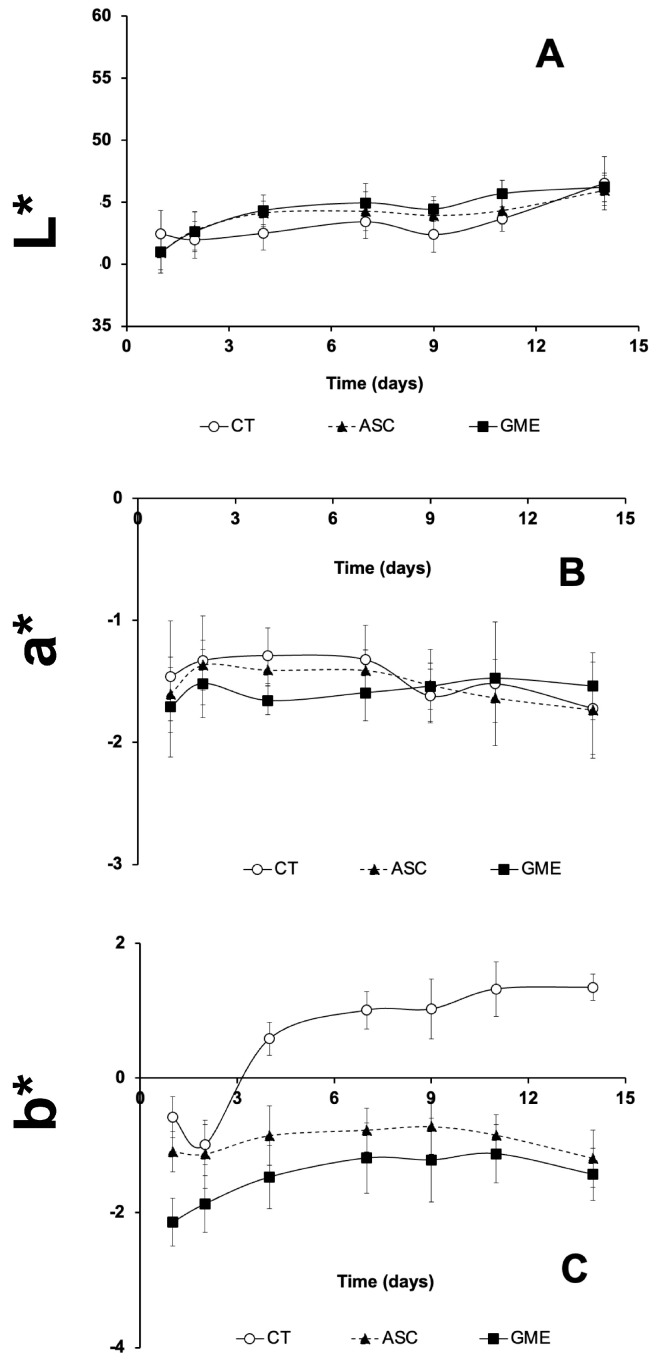
Postmortem changes in chromatic parameters ((**A**): lightness, L*; (**B**): redness/greenness, a*; (**C**): yellowness/blueness, b*) of muscle side of gilthead seabream fillets treated with the different experimental solutions during 14 days of cold storage at 4 °C. CT: control, ASC: ascorbic acid, GME: grape marc extract. Values are expressed as mean ± s.d. (n = 4 fillets per dietary treatment and sampling time).

The influence of the experimental extracts applied to gilthead seabream fillets on the chromatic parameters L* (lightness), a* (redness), and b* (yellowness) is summarized in Figure 5 and Figure 6, for skin and muscle sides, respectively.

Skin lightness (L*) decreased in all samples with increasing storage time, although roughly, GME fillets yielded the higher values for this parameter throughout the whole period. On the other hand, ASC fillets showed the lower values for L* up to 11 dpm. However, the differences in GME compared to CT were significant only at the beginning of the assay (1 dpm) and up to 7 dpm compared to the ASC batch.

As for a* values, all fillets showed a decreasing trend with storage time, from positive values (slightly reddish tones) to negative values (greenish color), regardless of the treatment (*p* ≤ 0.001). In general, the values of the GME treatment were lower than those of the rest of the other batches (ASC and CT) and these differences were significant at 1, 2, 7, and 14 dpm. On the other hand, the results of b* (Figure 5C) also showed a decreasing trend during the cold storage of the fillets, but this decrease due to the storage time was significant for the ASC and GME treatments, but not for the CT group. Regarding the effect of the additive, although all the fillets initially presented similar values, after 4 dpm, the values of the GME fillets were statistically lower (less yellow) than those of CT and ASC.

The chromatic parameters L* and b* of the muscle side of the fillets were influenced by the factors, storage time, additive, and their interaction, whereas a* parameter was only influenced by storage time. Muscle lightness (L*) increased with storage time (*p* ≤ 0.001) in all the experimental groups (Figure 6A). Considering the effect of the antioxidant solutions applied on the fillet surface, both ASC and GME batches showed statistically higher L* values compared to the untreated fillets (CT) at 4 and 9 dpm. When comparing the two additives, the GME fillets generally had higher values for this parameter, although the differences were not significant until 11 dpm.

No significant changes were observed due to storage time or treatment for the parameter a* (Figure 6B). However, antioxidant solutions induced specific changes at 4 and 7 dpm, with CT fillets showing lower values for this parameter.

Finally, the parameter b* (Figure 6C) showed a marked increase in the CT group due to the storage time, which was not as evident in the ASC and GME groups. Thus, the CT fillets showed positive values starting from 4 dpm, indicating a yellowish color, whereas the ASC and GME fillets showed slightly negative values close to zero throughout the trial, indicating neutrality (white color). The use of ASC or GME additives resulted in a significant decrease in b* value over the storage time compared to the CT group. When comparing the effect of both additives on this parameter, (GME) generally showed lower values than ASC, with the differences being statistically significant only at 1, 4, and 7 dpm.

## 4. Discussion

In recent years, the use of phytochemicals from agri-food industry by-products as sources of food additives has attracted research interest [1,2,14]. This is mainly because they are abundant, readily available, relatively inexpensive, and acceptable to consumers, as they do not originate from chemical synthesis. In addition, many of them are attributed to interesting bioactive properties, which allow the valorization of by-products, transforming them into biomass with an interesting added value [5]. In this context, viticulture by-products, not least grape marc, have aroused interest in the food, pharmaceutical, and cosmetic industries due to their high content of bioactive compounds, especially polyphenols, mainly owing to the beneficial effects of such compounds on human health [19,30,31]. **The grape marc resulting from white wine production, which is the marc used in this study, comes directly from the pressing of the grapes, without any further transformation, whereas the marc from red wine production undergoes a fermentation stage after pressing** [32]. This implies that white grape marc contains a high number of polyphenols and other bioactive compounds present in the original grapes, while red grape marc is worn out, as these bioactive compounds have been transferred to the wine.

The antioxidant activity of the grape extract is mainly related to the biological activity of polyphenols [33]. This was measured in GME (Table 1), together with total phenolics, and the results indicate that the grape extract used in our study has considerable antioxidant activity, as FRAP (ferric reducing antioxidant power) and DPPH (radical scavenging activity) values were even higher than those measured by Sáez et al. [34], who used different seaweed extracts as an antioxidant additive in rainbow trout fillets. For example, the antioxidant activity of GME exceeded that of *Arthrospira platensis*, the most notable extract in that study, whereas DPPH values were comparable, a fact that may well be attributed to the high polyphenol content of GME.

There is currently a need in the food industry to extend the catalog of additives available for fresh fish, preferably of natural origin rather than chemically synthesized. It is in this context that this research was carried out to evaluate GME extract as a potential additive for fresh fish fillets.

Lipid oxidation is one of the processes that most clearly reduces the shelf-life of fish, owing to the richness of fish lipids in n-3 PUFAs. These FAs are more sensitive to lipid oxidation than saturated lipids, a phenomenon that contributes to the appearance of unpleasant odors and flavors associated with fish spoilage [35], as well as increasing the levels of secondary compounds of lipid oxidation, such as malondialdehyde (MDA). This molecule is associated with the second phase of lipid autoxidation, in which peroxides are oxidized to ketones and aldehydes [36]. It is essential to measure the antioxidant activity of any extract with potential value as a food additive for fish. The increase in TBARS values over time can be attributed both to the partial dehydration of fish that occurs during refrigeration and, more importantly, to the oxidation of PUFAs [37,38]. In this context, GME extract applied to gilthead sea bream fillets was able to significantly inhibit lipid oxidation compared to untreated fillets (CT) throughout the experiment (14 dpm), as shown in Figure 2. For up to 7 d cold storage, GME and ASC, the additive authorized in the EU, caused comparable antioxidant effects, but from this time onward, the GME batch showed significantly lower TBARS levels than ASC fillets. The antioxidant properties of the treatments are reflected in the fact that the maximum limit set for human consumption of TBARS, expressed in MDA equivalents (8 mg kg^−1^ according to Erkan and Özden [39]), was approximately reached in the CT fillets at 11 dpm, whereas the ASC-treated fillets reached this value at the latest sampling time (14 dpm). Interestingly, the GME fillets showed even lower values at this time, and the maximum MDA limit mentioned above was not reached during the entire experimental period. It should be noted that this time is well beyond the normal commercial shelf life of fresh fish.

In this regard, the study by Li et al. [40] applied a grape seed extract (GSE) solution to fillets of the perciform *Channa argus* (snakehead fish) and evaluated its effect on muscle protein degradation. The results obtained showed that the GSE extract was able to reduce the rate of protein degradation by half compared to the untreated control group. The authors attributed the results to the antioxidant properties of the polyphenols (especially proanthocyanidins) present in the GSE, which have been previously described for grape seeds [41]. Guan et al. [42] found that an aqueous extract of grape seed, sage, and oregano applied to fish surimi, reduced lipid oxidation, and Pazos et al. [35] reported that GME extract on Atlantic mackerel (*Scomber scombrus*) reduced muscle lipid oxidation. Raeisi et al. [43] described that a carboxymethylcellulose coating solution containing grape seed extract reduced undesirable proteolysis and lipid oxidation reactions in rainbow trout muscle.

The shelf life and safety of seafood products are also directly affected by microbial spoilage [44], which affects not only their nutritional but also their hygienic quality. This is because psychrophilic microorganisms can grow on fish even at refrigeration temperatures. The results obtained in the present study indicate that the use of GME on gilthead seabream fillets significantly reduced the psychrophilic bacteria counts (Figure 1), with antimicrobial effects similar, and even superior, to those obtained with ASC. This trend was also described in the study of Hasani and Hasani [44], who obtained similar results using grape extract in common carp. Likewise, the use of GSE at 0.2% on fillets of channel catfish (*Ictalurus punctatus)* [38] and snakehead fish [40] effectively inhibited microbial growth. In line with this, Trošt et al. [45] demonstrated the high antimicrobial activity of grape marc extract against both Gram (+) and Gram (−) bacteria, attributing these results to the high flavonol content of the grape varieties used. Continuing with our results, at 9 dpm, the CT and ASC fillets almost reached the maximum consumption limit of 7 log CFU g^−1^ for fish [46], whereas in the GME batch, this value was not reached until 11 dpm, demonstrating an inhibitory effect on microbial growth. Zhao et al. [47] also found comparable antimicrobial effects in tilapia fillets treated with 0.5% GSE, which in contrast to the control group, remained within human consumption limits throughout a storage period of 12 days.

The pH is also an indicator of fillet quality, and its value is increasingly affected by time during cold storage, as observed in Figure 3A. This increase is mainly attributable to the formation of alkaline compounds and volatile basic components, such as ammonia and trimethylamine, as a result of muscle protein degradation. These products also originate from the metabolism of growing bacteria, and contribute to the deterioration of fish [40]. Thus, Fan et al. [37] applied a 0.2% tea polyphenol solution by immersion of silver carp fillets (*Hypophthalmichthys molitrix*), and they observed a delay in the increase in pH, which correlated with the inhibition of microbial growth. However, in our study, although a reduction in microbial growth was observed with the application of the additives compared to the control fillets (Figure 1), these differences were not reflected in lower pH values throughout the experimental period, even though the pH values measured in this study remained within the range characterizing fresh fish (6.0 to 6.5) and were below the upper acceptable limit of 6.8 to 7.0 [39] for all the experimental batches.

Water holding capacity (WHC) provides information on the amount of water the muscle can retain, and this parameter is directly related to juiciness, affecting the texture of the fillet, as low WHC values resulted in reduced muscle weight and a dry, soft, or inconsistent appearance [48,49]. Furthermore, it is a clear indicator of the denaturation of myofibrillar proteins, as they lose their ability to retain water, leading to the loss of intra- and extracellular water in the muscle [50]. In this study, the WHC values were slightly higher in the GME-treated samples during up to 4 dpm (significantly different only at 2 dpm), which could indicate a beneficial effect of the grape extract, rich in polyphenols, on the mechanical properties of the muscle. These polyphenols might have delayed the oxidative denaturation of proteins and the aggregation of myofibrillar proteins, allowing a stronger binding between these proteins and water [38,51]. This effect has also been reported in other studies where grape seed extract was found to increase WHC levels in common carp fillets [50] and catfish (*Ictalurus punctatus* [38]) fillets.

Postmortem muscle deterioration occurs during cold storage due to the relaxation of its structure mediated by endogenous proteolysis of myofibrillar proteins and connective tissue [47], which is reflected in a decrease in muscle consistency and firmness. This phenomenon, combined with microbial spoilage, ultimately leads to the softening of the fish fillet [34]. This was observed in all the batches studied, but it is noteworthy that fillets treated with additives (ASC and GME) showed higher firmness values, with significant differences from 7 dpm onward (Figure 4). These results suggest that the additives applied to the surface of the fillets significantly slowed down the above-mentioned deterioration processes. The same trend was observed for chewiness and gumminess parameters. Similar results were obtained after the application of tea polyphenols by Wang et al. [36], where fillets of Japanese sea bass (*Lateolabrax japonicus*) were immersed in an aqueous extract, which preserved their physical characteristics and freshness during cold storage. Zhao et al. [47] reported that coatings based on plant extract solutions (grape seed extract and green tea) mixed with chitosan and applied to fillets were able to slow down the deterioration of the textural parameters compared to untreated fillets.

Another attribute that directly influences the consumer’s decision to purchase fish is the color of both the skin and the muscular side of the fillet. Our results show that, in general, the skin side of the gilthead sea bream fillet after the application of the grape marc extract was brighter (significant differences for L* parameter at 1, 4, and 7 dpm), greener (significant differences for a* parameter at 1, 2, 7, and 14 dpm), and less yellowish (significant differences for b* parameter from 4 dpm) compared to the other fillets. Similarly, Sáez et al. [34] observed an increase in L* values in the skin of the fillets after the surface application of algae extracts (0.3% concentration) on rainbow trout fillets.

On the other hand, the L* and a* parameters of the muscle side of the fillets were not significantly modified by the antioxidant-enriched solutions. More specifically, at certain sampling times (4 and 7 dpm), the muscle of the fillets treated with additives had lower a* values (less reddish, more greenish) compared to the CT batch, especially in the case of the GME-treated fillets. This could be explained by the fact that GME-treated proteins develop a brown color as the pigments oxidize [20]. The same trend for the a* parameter in the muscle was described by Zhao et al. [47] with the surface application of aqueous grape seed extract (0.5%, *p*/*v*) on Asian sea bass (*Lates calcarifer*) fillets, which delayed the discoloration of the fillet compared to the CT during storage. In that study, these results were attributed to the influence of the original color of the extracts (red-purple) on these parameters, with the protection of the color being attributed to the antimicrobial and antioxidant capacity of the grape seed extract.

The increase in b* values in the fish muscle during cold storage, as seen in Figure 6C, is associated with higher levels of lipid oxidation, as described by Lin et al. [52] and Silva-Brito et al. [53]. Our results show that fillets treated with an ASC and GME coating had lower b* values throughout the study period, in contrast to the CT fillets, which tended to be yellowish (positive b* values). This effect confirms the antioxidant role of the two additives evaluated (ASC and, especially, GME), in line with the changes in lipid oxidation observed in Figure 2, which were able to prevent the increase in the b* parameter over time. There are conflicting results in the literature on this aspect. For example, the use of algal extracts (micro and macroalgae) and polyphenolic extracts based on tannins added at a level of 0.3% also resulted in less yellowing of rainbow trout fillets during cold storage, results that can be attributed to the lower levels of lipid oxidation observed for these treatments [34,54]. However, in the study by Zhao et al. [51], fillets treated with grape seed extract showed positive b* values and a more yellowish color. In this case, the authors attributed this to the influence of the original color of the extract, which might have masked the reduction in lipid oxidation.

Although the experimental determinations do not indicate any detrimental effect on the objective quality of the fillet attributable to the use of GME extract, a sensory panel analysis is still necessary to detect possible changes in the organoleptic attributes that could affect the consumers’ acceptability of the treated product.

## 5. Conclusions

The application of aqueous grape marc extract (GME) to the surface of gilthead seabream fillets as an additive resulted in a significant improvement in several quality parameters during cold storage, with an overall delay in spoilage. The extract was able to inhibit microbial growth, particularly in the early stages of storage, extending the shelf life of the fillets up to 11 days in cold storage, even exceeding the preservative effects of the ascorbic acid (ASC) treatment.

GME was also shown to be effective in reducing lipid oxidation, thereby preserving the freshness of the fillets during cold storage. This antioxidant effect is crucial for improving product quality and safety by delaying rancidity processes. GME also contributed to maintaining the textural parameters of the fillets, mainly firmness, suggesting a slowing down of structural deterioration, similar to the effect of ASC. Textural parameters are essential for consumer acceptance of fresh fish. Finally, the treatment with GME improved color parameters, namely an increase in fillet skin and muscle lightness (L*) and a decrease in yellowness (b* parameter), which together contribute to a more visually appealing product. Nevertheless, an expert sensory panel analysis is necessary to ascertain any possible impact on organoleptic attributes that could affect the acceptability of the product.

In conclusion, GME appears to be a viable alternative as a natural preservative for fresh fish, with benefits in terms of both shelf-life extension and preservation of sensory properties. Furthermore, its use is in line with the principles of the circular bioeconomy, promoting the reuse and valorization of agro-industrial by-products and contributing to the sustainability of the aquaculture industry.

## Figures and Tables

**Table 1:** Antioxidant activity of grape marc extract.

TPC ^1^	5.48 ± 0.17
FRAP ^2^	21.99 ± 0.57
DPPH ^3^	7.6 ± 0.75

^1^ TPC: total phenolic contents, expressed as mg of gallic acid equivalents mL^−1^. ^2^ FRAP: ferric reducing antioxidant power, expressed as μmol equivalent of Trolox mL^−1^. ^3^ DPPH: radical scavenging activity, expressed as μmol equivalents Trolox mL^−1^.

**Table 2:** Changes in texture profile analysis (TPA) parameters in seabream fillets during a 14 d cold storage (4 °C) period.

		Diets						
Parameters	dpm	CT	ASC	GME	*p*	Treat.	Time	Treat. × Time
Springiness (mm)	1	0.71 ± 0.02 ^1^	0.69 ± 0.02	0.71 ± 0.02 ^1^	0.547	0.249	≤0.001	0.099
	2	0.75 ± 0.01 ^123^	0.76 ± 0.04	0.74 ± 0.01 ^12^	0.919			
	4	0.79 ± 0.03 ^3^	0.75 ± 0.01	0.77 ± 0.01 ^2^	0.176			
	7	0.74 ± 0.01 ^12^	0.74 ± 0.01	0.74 ± 0.01 ^12^	0.882			
	9	0.79 ± 0.01 ^b,3^	0.77 ± 0.02 ^b^	0.71 ± 0.01 ^a,1^	≤0.001			
	11	0.76 ± 0.01 ^23^	0.72 ± 0.01	0.75 ± 0.02 ^12^	0.148			
	14	0.74 ± 0.01 ^12^	0.75 ± 0.02	0.78 ± 0.03 ^2^	0.425			
	*p*	0.006	0.064	0.041				
Cohesiveness	1	0.47 ± 0.01	0.46 ± 0.02	0.47 ± 0.01 ^12^	0.824	0.689	0.008	0.461
	2	0.47 ± 0.03	0.51 ± 0.01	0.45 ± 0.01 ^1^	0.166			
	4	0.47 ± 0.01 ^a^	0.45 ± 0.01 ^a^	0.50 ± 0.01 ^b,2^	0.011			
	7	0.47 ± 0.02	0.45 ± 0.02	0.46 ± 0.01 ^12^	0.810			
	9	0.50 ± 0.01	0.49 ± 0.02	0.50 ± 0.02 ^2^	0.783			
	11	0.48 ± 0.01	0.46 ± 0.02	0.44 ± 0.04 ^1^	0.596			
	14	0.51 ± 0.01	0.50 ± 0.01	0.51 ± 0.02 ^2^	0.737			
	*p*	0.435	0.135	0.057				
Gumminess (N mm^−2^)	1	8.25 ± 0.38 ^4^	7.94 ± 0.40 ^34^	8.05 ± 0.50 ^34^	0.876	≤0.001	≤0.001	0.595
	2	6.89 ± 1.14 ^234^	8.46 ± 0.38 ^4^	7.80 ± 0.32 ^234^	0.312			
	4	6.98 ± 0.19 ^a,34^	7.35 ± 0.37 ^a,234^	8.57 ± 0.33 ^b,4^	0.005			
	7	6.05 ± 0.31 ^123^	7.01 ± 0.32 ^123^	7.34 ± 0.60 ^234^	0.108			
	9	5.97 ± 0.30 ^123^	7.34 ± 0.71 ^234^	7.07 ± 0.33 ^123^	0.126			
	11	5.30 ± 0.23 ^1^	6.32 ± 0.52 ^12^	5.93 ± 0.47 ^1^	0.245			
	14	5.50 ± 0.33 ^12^	5.94 ± 0.58 ^1^	6.60 ± 0.45 ^12^	0.252			
	*p*	0.002	0.009	0.002				
Chewiness (N mm^−1^)	1	5.93 ± 0.42 ^3^	5.45 ± 0.32 ^123^	5.71 ± 0.38 ^23^	0.667	0.009	≤0.001	0.335
	2	5.15 ± 0.87 ^123^	6.43 ± 0.47 ^3^	5.80 ± 0.28 ^23^	0.325			
	4	5.54 ± 0.29 ^a,23^	5.48 ± 0.30 ^a,123^	6.65 ± 0.24 ^b,3^	0.014			
	7	4.45 ± 0.24 ^12^	5.23 ± 0.30 ^12^	5.42 ± 0.45 ^12^	0.120			
	9	4.71 ± 0.23 ^12^	5.69 ± 0.60 ^23^	5.03 ± 0.28 ^12^	0.233			
	11	4.04 ± 0.16 ^1^	4.59 ± 0.38 ^12^	4.47 ± 0.44 ^1^	0.506			
	14	4.08 ± 0.25 ^1^	4.42 ± 0.38 ^1^	5.10 ± 0.30 ^12^	0.085			
	*p*	0.012	0.019	0.004				
Resilience (N mm^−1^)	1	0.21 ± 0.01 ^1^	0.20 ± 0.01 ^1^	0.22 ± 0.00 ^12^	0.353	0.036	0.009	≤0.001
	2	0.23 ± 0.01 ^a,12^	0.27 ± 0.02 ^b,2^	0.21 ± 0.01 ^a,1^	0.005			
	4	0.23 ± 0.01 ^b,12^	0.20 ± 0.01 ^a,1^	0.26 ± 0.01 ^c,3^	≤0.001			
	7	0.23 ± 0.01 ^12^	0.22 ± 0.01 ^1^	0.21 ± 0.01 ^1^	0.337			
	9	0.24 ± 0.01 ^2^	0.23 ± 0.01 ^1^	0.23 ± 0.01 ^123^	0.608			
	11	0.25 ± 0.01 ^b,2^	0.20 ± 0.01 ^a,1^	0.22 ± 0.02 ^ab,12^	0.017			
	14	0.24 ± 0.01 ^2^	0.22 ± 0.01 ^1^	0.24 ± 0.01 ^23^	0.075			
	*p*	0.039	≤0.001	0.020				

Treatments: CT: control. ASC: ascorbic acid solution. GME: grape marc extract solution. Superscript letters indicate differences attributable to treatments within each storage time. Superscript numbers indicate differences attributable to storage time within each treatment (*p* < 0.05). The effect of the categorical variables “antioxidant treatment” (Treat.) and “storage time” (Time), as well as their interactions (Treat. × Time), were determined by fitting a generalized linear statistical model (GLM). Values are expressed as mean ± sd (n = 4 fillets per dietary treatment and sampling time). dpm: days postmortem. n.s.: not significant.

## Data Availability

The original contributions presented in the study are included in the article, further inquiries can be directed to the corresponding author.
